# People management: from the intuitive process to People Analytics

**DOI:** 10.1590/S1679-45082018CE4398

**Published:** 2018-06-15

**Authors:** Quevellin Alves dos Santos

**Affiliations:** Universidade Federal de Alfenas, Alfenas, MG, Brazil

Dear editor,

Francis Bacon proposed in 1620 that scientific knowledge should be not only based on intuitive hypothesis, which is eventually limited to establish a premise before an experience, and, therefore, restrict thinking in a way that correlate with this principle. Currently, Big Data has promoted shifts on previous paradigms. New studies are based on network through massive amount of data and even less based only on intuitions.^(^ ^1^ ^)^ Big Data applied to people management is called “People Analytics” and it comprises in a survey and analytical process of large amount of data from workers within an organization in order to support the decision process related with people management.^(^ ^2^ ^)^ Benefits of people analytics include the promotion of more strategic people management based on objective data. Health professionals profile requires personal and professional characteristics along with today´s health development that includes technological innovation and evidence-based practice.^(^ ^3^ ^)^ This transformation occurs both at organizational and technological level and it can bring efficient results for companies and might be greater than results expected by a number of organizations.^(^ ^2^ ^)^
